# Physical forces guide curvature sensing and cell migration mode bifurcating

**DOI:** 10.1093/pnasnexus/pgad237

**Published:** 2023-08-01

**Authors:** Luyi Feng, Tiankai Zhao, Hongmei Xu, Xuechen Shi, Changhao Li, K Jimmy Hsia, Sulin Zhang

**Affiliations:** Department of Engineering Science and Mechanics, Pennsylvania State University, University Park, PA 16802, USA; Department of Engineering Science and Mechanics, Pennsylvania State University, University Park, PA 16802, USA; School of Mechanical and Aerospace Engineering, Nanyang Technological University, Singapore 639798, Singapore; Department of Biomedical Engineering, Pennsylvania State University, University Park, PA 16802, USA; Department of Engineering Science and Mechanics, Pennsylvania State University, University Park, PA 16802, USA; School of Mechanical and Aerospace Engineering, Nanyang Technological University, Singapore 639798, Singapore; School of Chemistry, Chemical Engineering and Biotechnology, Nanyang Technological University, Singapore 639798, Singapore; Department of Engineering Science and Mechanics, Pennsylvania State University, University Park, PA 16802, USA; Department of Biomedical Engineering, Pennsylvania State University, University Park, PA 16802, USA; Department of Materials Science and Engineering, Pennsylvania State University, University Park, PA 16802, USA

**Keywords:** surface tension, molecular flow, cell migration, phase-field modeling

## Abstract

The ability of cells to sense and adapt to curvy topographical features has been implicated in organ morphogenesis, tissue repair, and tumor metastasis. However, how individual cells or multicellular assemblies sense and differentiate curvatures remains elusive. Here, we reveal a curvature sensing mechanism in which surface tension can selectively activate either actin or integrin flows, leading to bifurcating cell migration modes: focal adhesion formation that enables cell crawling at convex front edges and actin cable assembly that pulls cells forward at concave front edges. The molecular flows and curved front morphogenesis are sustained by coordinated cellular tension generation and transmission. We track the molecular flows and mechanical force transduction pathways by a phase-field model, which predicts that multicellular curvature sensing is more efficient than individual cells, suggesting collective intelligence of cells. The unique ability of cells in curvature sensing and migration mode bifurcating may offer insights into emergent collective patterns and functions of living active systems at different length scales.

**Significance Statement**

Curved surfaces and edges are ubiquitous in living organisms. The ability of cells to sense and adapt to different curvatures facilitates organ morphogenesis, tissue repair, and tumor metastasis. However, how cells sense curvatures and adopt different migration modes is only beginning to be understood. Here, we reveal that curvature sensing and subsequent cell migration mode branching involve coordinated physical force generation and transmission, molecular flow activation and sustentation, and curved front morphogenesis. Our studies point to a general principle of physical force–activated molecular assembly in directional cell migration. The unique ability of cells in sensing curvature variations and branching migration modes contributes to the robustness of cell migration.

## Introduction

Directional cell migration underlies a broad range of phenomena in tissue and organ morphogenesis, wound healing, cancer metastasis, etc. Much has been known about cell migration by following concentration gradient of soluble cues in chemotaxis (1–7) and stiffness gradient of extracellular matrices in durotaxis (8–12). In vivo, the microenvironments of cells span an impressive range of curvy surfaces and edges (13–16), suggesting that cells may be able to sense and adapt to curvatures. At molecular scale, transmembrane proteins of specific shapes in locally curved membrane domains diffuse and aggregate, exhibiting a “curve-to-attract” phenomenon (17, 18). At cellular levels, T cells preferentially migrate along concave microgrooves and endothelial cells elongate and align in response to the curvature of a rod assay (19). At multicellular and tissue level, epithelial cells bridge nonadhesive gaps in a curvature-dependent manner (15) and cancerous human colon carcinoma cells at convex edges are more prone to metastasis than those at concave edges (20). Despite the abundant relevant experimental evidence at different length scales and dimensions, how cells sense, differentiate, and adapt to curvatures have remained unclear.

Studies on durotaxis (21, 22) and chemotaxis (23) evidenced that directional cell migration involves two essential steps: an activation step involving cell polarization and the subsequent cell movement. These two steps occur at different time scales: short-time scale for cell polarization (hereafter referring to as the polarization time scale) (24) and long-time scale for cell movement (the migration time scale) (25). On the migration time scale (tens of minutes to hours), cell changes its positions, accompanied by apparent morphological changes; on the polarization time scale (seconds to minutes), cell morphology remains nearly unchanged, while endogenous molecular flows are activated to assemble cell migratory structures for cell polarization. Although cell migration on the long-time scale has been experimentally (15, 25, 26) and numerically (27–33) investigated, how cell achieves its migration directionality, i.e. the activation mechanism, remains poorly understood.

It has been widely reported that cells on a flat substrate can adapt to two distinct migration modes: lamellipodial protrusion-based cell crawling for convexly curved front (34, 35) and actin cable-based contraction for concavely curved front (36–38). How cells sense the curved front edges and activate the corresponding migration mode remains elusive. Here, we reveal that surface tension at the curved front edges can activate branching molecular flows: actin flow for concavely front edges and integrin flow for convexly front edges. The molecular flows facilitate curved front morphogenesis: actin cable assembling at the concave front and focal adhesion formation at the convex front. Coordinated cellular force generation and transmission, molecular flows, and migratory structure assembly form chemomechanical feedback loops that evolve to bifurcating migration modes. A multi-phase-field model is formulated to follow the feedback loops. In addition to highlighting the critical roles of physical forces in activation and sustentation of the molecular flows, our model predicts high efficiency of multicellular curvature sensing and adaption compared with single cells.

### Cell migration mode precursors at curved fronts

**Fig. 1.**
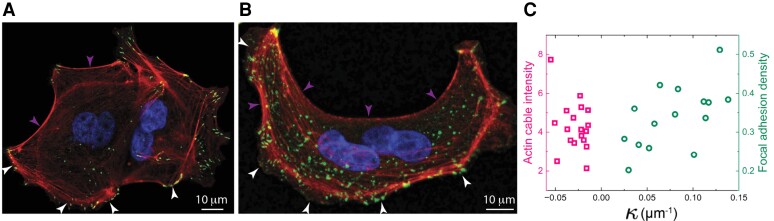
(A, B) Fluorescent images of the bifurcating morphologies at the curved edges of (MDCK) epithelial cell colonies: focal adhesion formation at convex edges indicated by vinculin staining (green, marked by bright arrows) and actin cable formation at the concave edges indicated by actin staining (red, marked by dim arrows). Nuclei were stained by blue. (C) Curvature-dependent densities of actin stress fibers and focal adhesions show asymmetric sensitivity of the cells to the positive and negative curvatures.

To see how cells sense and adapt to curvatures, Madin–Darby canine kidney (MDCK) epithelial cells were seeded on a flat substrate, forming multicellular colonies that exhibited both positively and negatively curved front edges (Fig. 1A and B). Depending on the sign of curvatures, distinctive curved front subcellular microstructures emerge at concavely curved (negative curvature) front actin cables that were self-assembled, while at convexly curved (positive curvature) front focal adhesion complexes that were formed. These curved front subcellular microstructures are precursors of specific cell migration modes: actin cables contract to pull the cells forward, which often occurs when cells invade nonadhesive gaps (37–43), while lamellipodia/filopodia protrude from the focal adhesion complexes for cell crawling on the substrate (44, 45). In the absence of a central organizing system, cells act only on their perception of local conditions—curvatures—to assemble distinctive curved front structures for migration. This indicates that cells can sense and distinguish positive and negative curvatures and adopt either lamellipodium-based cell crawling or actin cable contraction for cell migration, as observed in previous experiments (25).

To further appreciate cells’ ability in curvature sensing, we mapped out the densities of the actin stress fibers at concavely curved fronts and focal adhesions at the convexly curved fronts of the MDCK epithelial cell colonies (Fig. 1C). Here, we only plot the dominant molecular species at the corresponding curved edges, i.e. actin fibers at concave edges and focal adhesions at convex edges. The contrast at the curved front in the densities of the dominant and secondary molecular species is clearly visible from fluorescent images (Fig. S1) and quantitatively analyzed previously (36, 46, 47). With increasing magnitude of curvature, focal adhesion density at convexly curved fronts gradually increases, whereas actin stress fiber density abruptly arises at a very small concave curvature. The different slopes in the density profiles indicate that the cells are more sensitive to the concave curvatures than the convex ones (Fig. 1C). This asymmetric curvature sensing mechanism, which has so far remained elusive, may underlie curved front morphogenetic processes at the subcellular to cellular scale.

## Results

### Surface tension at the curved front activates branching molecular flows

To see how a cell senses curved front edges, we consider a cell seeding on a flat surface displaying a curved front. Since cell shape change occurs at a much longer time scale than that of the intracellular molecular flows, cell shape in our analysis remains nearly unchanged. Under this static background, we consider the internal dynamics of the molecular flows in response to the curved front. Capillary force, $\kappa \gamma_{0}$, arises at the curved front (48), where κ is the local curvature and $\gamma_{0}$ is the line tension of the front edge. The reference condition is naturally set as a straight front edge (κ=0) across which the pressure inside ($P_{\mathrm{in}}$) and outside ($P_{\mathrm{out}}$) of the cell is balanced, denoted by $P_{0}$. The pressure in the interior of the cell at the reference state is also assumed to be uniform ($P_{0}$). When the cell front changes from a straight to a curved edge, the capillary force changes both its magnitude and direction, which modifies the inner pressure proximal to the curved edge ($P_{\mathrm{in},\;\;\mathrm{near}}$), while the inner pressure far from the curved edge ($P_{\mathrm{in},\;\;\mathrm{far}}=P_{0}$) and the pressure outside the cell ($P_{\mathrm{out}}=P_{0}$) remain unchanged. A pressure difference across the curved edge arises according to the Young–Laplace law:


(1) $$\Delta P=P_{\mathrm{in},\mathrm{near}}-P_{\mathrm{out}}=P_{\mathrm{in},\mathrm{near}}-P_{\mathrm{in},\mathrm{far}}=\kappa \gamma_{0}$$

**Fig. 2.**
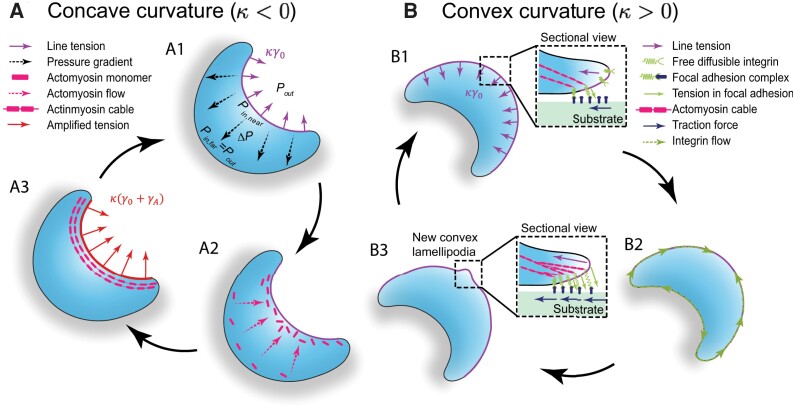
Feedback loops of cellular force generation, molecular flows, and curved front motif assembling in curvature sensing. (A) At a concave front edge (purple lines), line tension generates a far-to-near pressure gradient (black dashed arrows, A1), which drives the flows of actomyosin motors and actin monomers to the curved front (pink dashed arrows, A2), leading to the actin cable formation and enhanced line tension (red arrows, A3). Since actin cable contracts in the same direction as line tension, a positive feedback reinforces this process. (B) At a convex front edge, line tension points toward the cell (B1). This curvature-induced retraction stretches focal adhesion complexes, generating tension inside them to counteract the retraction at the front. (B1, inset figure) Tension in the focal adhesions attracts integrin flow (marked by green dashed arrows) toward the curved front (B2), leading to focal adhesion growth, traction force reinforcement, and lamellipodial protrusion (B3). The tension in the focal adhesions, the integrin flow, and focal adhesion assembly form another positive feedback, leading to focal adhesion maturation.

The same pressure difference is established in the cell cytosol from near to far from the curved edge. According to the Gibbs–Thomson effect, a chemical potential gradient is created within cell cytosol,


(2) $$\Delta \mu_{0}=\Omega \kappa \gamma_{0}=\Omega \Delta P$$

which drives molecular diffusion, where Ω is the molar volume of the diffusive molecules, including actomyosin motors and actin monomers. For a concavely curved edge (κ<0), the chemical potential gradient ($\Delta \mu_{0}<0$) activates anterograde molecular flows toward the curved front (Fig. 2A1 and A2), whereas for a convexly curved edge (κ>0), the chemical potential gradient ($\Delta \mu_{0}>0$) generates retrograde flow (Fig. 2B1). The curvature-dependent actin flow directions are consistent with the previous illumination microscopy observations (25, 49), suggesting that surface tension–activated molecular flow is a key step in curvature sensing of cells.

### Migration mode bifurcation involves chemomechanical feedback loops

We next articulate how the activated molecular flows lead to different migration modes. Based on previous experimental observations (15, 20, 25, 50) and theoretical analyses (35, 51), we propose two chemomechanical feedback loops in the curvature sensing process. At a concavely curved cell front (κ<0), the chemical potential gradient arising from the pressure difference (Fig. 2A1) drives the transport and accumulation of actomyosin motors and actin monomers toward the front (Fig. 2A2). Such an anterograde flow of actins has experimentally been observed in previous works (15, 25). Line tension acting on the actins decreases the chemical potentials of actin monomers (52, 53), promoting self-assembly of actin cable (15, 43, 54) (Figs. 1 and 2A3). Actomyosin motors actively contract the actin cable along the curved edge, leading to an amplified tension $\kappa (\gamma_{0}+\gamma_{A})$ at the curved edge, where $\gamma_{A}$ denotes active contraction of the actin cable. The increased tension further lowers the chemical potential of the polymerized actins (55), corresponding to an increased chemical potential gradient $\Delta \mu =\Omega \kappa (\gamma_{0}+\gamma_{A})$ that up-regulates the active flow of motor proteins and actin monomers. The tension along the curved edge, actomyosin flow, and actin cable formation thus form a positive feedback, thickening and empowering the actin cable at the concave front. The contractile actin cable resembles a purse string, pulling the cells forward.

At a convexly curved front, actin network assembly is suppressed since the retrograde flow depletes actin monomers and motor proteins at the front (Fig. 1). However, line tension tends to retract the leading edge and the retraction force may be transmitted to the transient focal adhesion complexes (Fig. 2B1), generating tension inside the complexes (Fig. 2B1, inset figure). Integrins in the focal adhesion complexes sustain tension, whereas freely diffusible integrins on the cell membrane are in the tension-free state, creating a tensional gradient in integrins. Tension in focal adhesions lowers the chemical potential of the integrins (56, 57), up-regulating integrin clustering through membrane-mediated diffusion from the rear to the cell front (50–52) (Fig. 2B2). This directional diffusion of integrins toward the convex front and subsequent assembly of focal adhesion points therein (Fig. 2B3) reinforce the traction force, setting the cells in the crawling mode, as supported by previous experiments (50). The tensional gradient, integrin flow, and focal adhesion formation form another positive feedback, leading to focal adhesion growth and maturation. Lamellipodia or filopodia may then extrude from the focal adhesion points, facilitating cell crawling over the substrates.

### A phase-field model of migration mode bifurcating

To quantify the curvature sensing mechanism, we introduce a biophysics-based phase-field model to track the molecular flows, cellular force generation and transmission, and curved front migration bifurcation. It should be emphasized that our study focuses on the short-time cell activation step at an infinitesimal deformation and nearly fixed cell shape whose change occurs at a much longer time scale; subsequent cell migration and shape changes are not modeled. We model the spatiotemporal dynamics of three phase fields in 2D space x and time *t*: the displacement field of a multicellular monolayer u(x,t), the density fields of actomyosin motor proteins c(x,t) and the integrins ρ(x,t) (with 0≤c;ρ≤1). We assume codiffusion and colocalization of actin monomers and motor proteins to form contractile actin network. These three phase variables formulate a minimal model, where the displacement field characterizes the cellular force transmission, while the density fields infer the assembly of the cellular force generators and switchers, i.e. actin stress fibers and focal adhesions. In our minimal model, the dynamics of two density fields capture the precursor of two migration modes: actomyosin motor proteins c(x,t) at concave front for purse string–like contraction and integrins ρ(x,t) at convex front for cell crawling. We adopt focal adhesions as the precursor of lamellipodium-mediated cell migration. Lamellipodial protrusion involves a complex molecular signaling pathway and multiple molecular participants such as the ARP2/3 protein (58–61) and integrins. Adopting focal adhesions as the precursor offers more convenience in our cell migration activation model, since focal adhesion complexes more closely tie to cellular force transduction and molecular flows in the activation step. Within the phase-field framework, we construct a free energy functional of the cell–substrate system:


(3) $$F(c;\rho ;u)=\int_{A}\;[\;f_{g}(\nabla c)+f_{\mathrm{ch}}^{c}(c;u)+f_{\mathrm{ch}}^{\rho }(\rho ;u)+f_{\mathrm{el}}(c;u)]dA+\int_{\partial A}\;\gamma_{0}d\ell$$

Here, $f_{g}$, $f_{\mathrm{el}}(c;u)$, $f_{\mathrm{ch}}^{c}(c;u)$, and $f_{\mathrm{ch}}^{\rho }(\rho ;u)$ represent the gradient energy density, the elastic energy density, the chemical energies of motor proteins and integrins, respectively. The last term is the surface energy acting on the front edge. For simplicity, we assume that the substrate is much stiffer than the cell monolayer and its strain energy is neglected. The chemical energies of actomyosin and integrins involve entropic penalty for molecular aggregation and tension-induced enthalpy, consistent with experimental observations of actin cable formation (15, 36) and integrin clustering (49, 62, 63). Detailed expressions of the free energies are shown in the Supplementary material.

The relatively shorter time scale of the mechanical process ensures mechanical equilibrium of the cell monolayer at any time by δF/δu=0, giving rise to


(4) ∇⋅(hσ)−T=0,

where *h* is the thickness of the cell monolayer, σ is cell stress, and T is the tractions between substrate and colony. A traction boundary condition, $\sigma \cdot n=-\kappa \gamma_{0}/hn$, is imposed at the curved front edge with an outward normal n (64).

The chemical potential gradient $\nabla \mu_{c}$ drives the diffusion of the motor proteins: $j_{c}=-M_{c}\nabla \mu_{c}$, where $M_{c}$ is the mobility of the motor proteins. Conservation of motor proteins leads to the Cahn–Hilliard type diffusion equation:


(5) $$\frac{\partial c}{\partial t}=-\nabla \cdot j_{c}=\nabla \cdot (M_{c}\nabla \mu_{c}).$$

The integrin receptors on adherent cells exist in two distinct states: freely diffusible within the cell membrane or binding with the ligands to form ligand–receptor pairs. We treat the density of the bound integrins in the focal adhesions as a nonconserved phase variable, governed by the Allen–Cahn type equation:


(6) $$\frac{\partial \rho }{\partial t}=-M_{\rho }\mu_{\rho },$$

where $\;M_{\rho }$ is the mobility of the integrins on the cell membrane.

The mechanical stress field is coupled bidirectionally with the density fields. First of all, accumulation of the density fields implicates stress generation and transmission. The traction force T sustained in focal adhesion points depends on the local density of the integrins (ρ) bound to the ligands, T=Nρku, where *N* is the site number per unit area for integrins (35) and the ligand–receptor pairs are treated as linear springs with a spring constant *k*. Within the cytosol, cells contract by powering the actomyosin motors. The contractile strain generated by the motor proteins can be expressed as: $\varepsilon_{ij}^{c}(c;u)=-(\varepsilon_{0}+\beta c)\delta_{ij}$, where $\varepsilon_{0}$ is the ambient volumetric contraction and *β* is the contractability coefficient of the motor proteins (53, 65).

The total strain of the cell is the sum of the elastic strain and the contractile strain: $\varepsilon_{ij}^{\mathrm{tot}}(c;u)=\varepsilon_{ij}^{\mathrm{el}}(u)+\varepsilon_{ij}^{c}(c).$ The contractile strain sets a stress-free condition, whereas the elastic strain is related to the cell stress σ through the constitutive relation, treating the cell sheet as an elastic medium with Poisson's ratio ν and Young's modulus *Y*.

Reciprocally, the stress field drives molecular flows. The chemical potential of the motor proteins has a chemical and mechanical component: $\mu_{c}=\frac{\delta F}{\delta c}=\mu_{c}^{\mathrm{ch}}+\mu_{c}^{\sigma }$. While $\mu_{c}^{\mathrm{ch}}$ accounts for the local concentration of the motor proteins, $\mu_{c}^{\sigma }=h\beta \sigma_{\mathrm{kk}}-\alpha \sigma_{1}$ for the local stress state (see the Supplementary material), where $\sigma_{1}$ is the first principal stress (35, 52, 53), i.e. the contraction direction of the stress fibers. The term $-\alpha \sigma_{1}$ ensures that tension in the stress fibers attracts while compression repels motor proteins, where α is a coefficient. Similarly, the chemical potential of the integrins also consists of a chemical and a mechanical component: $\mu_{\rho }=\delta F/\delta \rho =\mu_{\rho }^{\mathrm{ch}}+\mu_{\rho }^{T}$. The chemical component $\mu_{\rho }^{\mathrm{ch}}$ accounts for the concentration of the integrins, while the mechanical component $\mu_{\rho }^{T}=-\frac{1}{2}T^{2}/k$ for the mechanoactiveness of focal adhesions: tension sustained on integrins lowers the chemical potential, activating integrin clustering (35, 51–53, 56, 57) (see the Supplementary material).

Solving the mechanical equilibrium condition (Eq. 4) and the two kinetics equations (Eqs. 5 and 6) simultaneously obtains the cellular force pattern, polarized actin network, and focal adhesion patterns in space and time. Here, we choose a cohesive multicellular colony with a flower-like shape as our simulation domain (Fig. S2). The flower shape exhibits concave, convex, and transition curved front edges, allowing simultaneous consideration of all the possible cases. We define a characteristic length scale of the cells $l_{0}=\sqrt{A}$, where *A* is the spreading area of the cell, and set a characteristic time scale for positive and negative curvature sensing to be $\tau_{+}=A/M_{c}$ and $\tau_{-}=A/M_{\rho }$, respectively.

### Coordinated cellular force patterning, molecular flows, and curved front morphogenesis

**Fig. 3.**
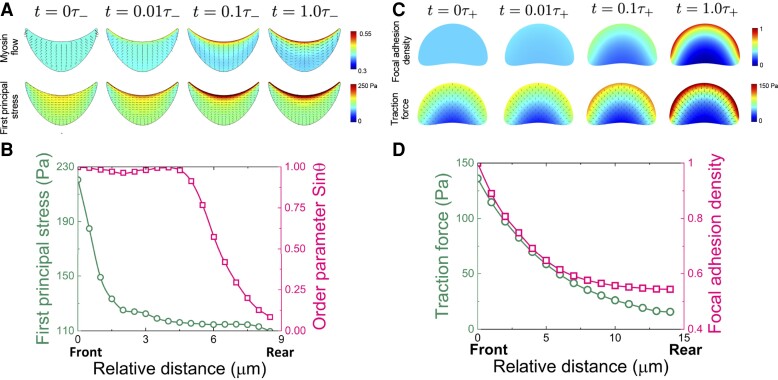
Simulated curvature sensing and migration mode bifurcation show cellular force–driven branching flows, leading to bifurcating assembly of actin cables (A, B) and focal adhesions (C, D) at the concavely and convexly curved fronts, respectively. (A) Concave front edge induces anterograde flow of actomyosin motors, generating actin cables at the curved front, indicated by the first principal stress profile. (B) The front–rear anisotropic distributions of the first principal stress and orientation order parameter of the stress fibers at the steady state. (C) Convex front edge generates retrograde flow of integrins and assembly of focal adhesion points, consistent with traction force profile. (D) The front–rear anisotropic distributions of the traction force and focal adhesion density at the steady state.

To quantify the branching feedback loops activated by surface tension, we now turn to the phase-field simulations of curved front morphogenesis by initializing our model with uniform density fields and the stress-free reference condition (35). Within the short activation time scale, the deformation field evolves under the nearly fixed front geometry. Our simulations reveal intimate coupling between curved front morphogenesis and cellular force patterns through the branching molecular flows. The simulated dynamics follow very well the feedback loops and naturally bifurcate to actin fiber assembly (Fig. 3A and B) at concavely curved front and focal adhesion formation (Fig. 3C and D) at convexly curved front. In addition, the simulated curve front morphogenesis resembles the experimental mappings in Fig. 1.

At concave front edge, such chemomechanical coupling is initiated by the line tension–induced pressure gradient (Fig. S3A) and proceeds by the polarized actin organization and concomitant anisotropic stress generation (Fig. 3A and B). While the active contractility drives the anterograde flow of actomyosin motors, the chemical composition gradient due to continuous accumulation of the motor proteins repels the flow. To show the active alignment of actin stress fibers along the convex front edge, we plot the direction of the first principal stress as the orientation order parameter of the stress fiber (66) α=sinθ at the steady state, where θ is the angle of the first principal stress with respect to the radial direction. The actin stress fibers transversely orient along the curved edge (α approaches 1), but radially at the rear (α approaches 0), as shown in Fig. 3B. Such chemical and stress anisotropies are consistent with the experimental observations (25). In contrast, integrin flow and accumulation are inactive and traction force pattern is nearly unchanged, indicating that cell migration through lamellipodia extension is suppressed near the concave edges (Fig. S3B and C).

At the convexly curved front, line tension activates retrograde flow of the motor proteins (Fig. S4A). To balance the line tension, cells develop focal adhesion complexes at the curved front and generate traction to the substrate. The tensional gradient drives the integrin flow to the curved front, while the chemical composition gradient due to the accumulation of integrins repels the flow (Fig. 3C). The accumulation of focal adhesion points at the curved front indicates anisotropic traction profile, breaking front–rear symmetry (25) (Fig. 3C and D). On the other hand, the actomyosin flow is directed to the rear, resulting in cell stress anisotropy opposite to the case in concavely curved front (Fig. S4B).

Since the surface tension triggers the bifurcating modes of migration, the resulting curved front morphologies and cellular force patterns depend on the front–edge curvature, but weakly on the back–side shape of the cells under a fixed cell spreading area (Fig. S5). At a curvature-varying edge of a single cell, our simulations show that morphogenetic flows of motor proteins and integrins along both radial and transverse directions are activated (Fig. S6), breaking both front–rear and left–right symmetries and leading to simultaneous assembly of actin cables and focal adhesion at the front.

### Long-range intercellular force transmission enables collective sensing

We next explore whether cells acquire collective ability of curvature sensing. Collective cell behavior requires cell–cell communication (9, 67), enabled by either diffusion of soluble cues (68) or chemomechanical switchers such as E-cadherin junctions (8, 69). It was previously found that collective durotaxis arises from long-range transmission of intercellular forces, where a multicellular colony can sense weak stiffness gradient of the substrate but isolated single cell cannot (8). Therefore, knocking down intercellular junctions may effectively suppress collective behaviors (69). Inspired by the biophysical origin of collective durotaxis (8), here, we present a simplified model to examine whether collective curvature sensing occurs. In this model, we use the stiffness of the cell–cell adherens junctions, denoted by τ (Pa/mm), to control intercellular interactions (see the Supplementary material). A vanishing τ corresponds to a fully blocked intercellular interaction and hence a weak collectiveness and isolated cells, while a large τ corresponds to efficient intercellular force transmission across the cells and hence strong collectiveness of a multicellular assembly. The efficiency of collective curvature sensing can be measured by the self-assembly of the migratory structures at the curved front, i.e. actin cable for the concavely curved front and focal adhesion for the convexly curved front. Alternatively, the generated cellular forces, denoted by ω can be used as the measures of curvature sensing ability, where ω=|T| for κ>0 and $\omega =\sigma_{I}$ for κ<0.

**Fig. 4.**
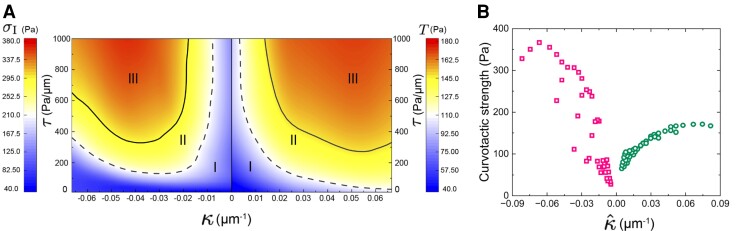
(A) Phase diagram of collective curvature sensing of cells. For positive/negative curvatures, the average extracellular traction/intracellular contraction at the curved front is used as a measure of the curvature sensing ability. Three phases, weak (I), moderate (II), and strong (III) curvature sensing strength, are identified. (B) The slopes of the ω–$\hat{\kappa }$ curves in simulation indicate much higher curvature sensitivity at negative curvatures than at positive curvatures, which corresponds to the asymmetric sensitivity in the experiments.

Our simulation results allow us to create a phase diagram of sensing ability in the plane of intercellular stiffness and curvature, as shown in Fig. 4A. The phase diagram identifies three regions, and the phasic profiles for positive and negative curvatures share the same trends. In phase I, either τ or |κ| is small, giving rise to a weak sensing ability. In phase III, both τ and |κ| are large, corresponding to a very high curvature sensing ability. An intermediate phase (II) also exists with a moderate ability. Our simulation results show that increasing the stiffness of intercellular adherens junctions enhances cells’ ability of force generation at the curved front for both positive and negative curvatures, demonstrating collective sensing ability of cells.

### Curvature sensitivity is asymmetric to positive and negative curvatures

Though cells appear to be sensitive and adaptive to both positive and negative curvatures, the markedly higher contraction ($\sigma_{I}$) in actin stress fibers than the traction in the focal adhesions (*T*) (Fig. 3) suggests asymmetric sensitivity to the sign of curvatures, consistent with the experimental observation that cells are more sensitive to the concave curvature than the convex front (Fig. 1C). We then define an effective curvature for collective cell sensing $\hat{\kappa }=\mathrm{sign}(\kappa )\sqrt{|\kappa |\tau /Y}$ and plot the curvature sensing strengths at positive (*T*) and negative ($\sigma_{I}$) curvatures (Fig. 4B). Our simulation data show a much steeper slope in the ω–$\hat{\kappa }$ curve for $\hat{\kappa }<0$ than that for $\hat{\kappa }>0$, which verifies the asymmetric curvature sensitivity of cells to the sign of curvatures. This asymmetric sensitivity partially explains the predominant flow rate of actins at the concave fronts upon activation (25).

## Discussion

Our theoretical and phase-field models link three spatiotemporal fields in curvature sensing and cell migration mode bifurcating: cellular forces to drive the molecular flows of motor proteins and integrins, aggregation of motor proteins to constitute actin cables at concavely curved front, and accumulation of integrins to form focal adhesion points at convexly curved front. The models capture the branching feedback loops in the initial mode activation step of cell migration, which involve interdependent subcellular processes including cellular force generation and transmission, molecular flows, and curved front morphogenesis. Activation of morphogenetic flows by capillary force constitutes a key step in curvature sensing. While the magnitude of capillary force determines whether a specific morphogenetic flow can be activated, the direction of capillary force leads to flow branching and migration mode bifurcating. Such a sensing and activation mechanism based on physical forces may be general at different length scales and dimensions. Thus, the mode activation step provides a basis for an extended model for cell migration and dynamic cell shape evolution in normal morphogenesis, tissue repair, and tumor metastasis (20, 26, 36, 43, 70, 71) under large time scale and large deformation. Our curvature sensing model can serve as a valuable initial step toward developing a framework for 3D cases, where cells sense and adapt to curved substrate surfaces (18, 69, 72–74). Indeed, significant modifications on assumptions and energetic formulation from the current 2D model are required. Nevertheless, our findings of surface tension–activated morphogenetic flows manifest the ability of cells to sense and respond to geomechanical cues in their environments.

Beyond the initial migration mode activation processes, cells demonstrate a remarkable capacity for spontaneous self-assembly of migratory entities, i.e. focal adhesions and actin cables, through stigmergic interactions. In the absence of a central organizing system, such curved front self-assembly processes are sustained by the generation and transmission of active tension. Indeed, tensional forces generated either in actin fibers or focal adhesions lay down a chemical potential gradient that drives the molecular flows toward targeted migration modes. Inspired by the shear flow in fluid dynamics, we term the molecular flows as “tensional flows” since they are activated by surface tension and sustained by active tension. Such tensional flow enabled curved front morphogenesis stands in stark contrast to the strategies by materials scientists in fabricating complex structures, where forces required for synthesis are often externally applied, rather than emerge from the structures being synthesized. This also highlights the major difference between passive systems and living active systems: while the former operates at or near thermodynamic equilibrium, the latter lives at nonequilibrium states driven by active forces. By understanding the ability of cells to sense their geomechanical cues and respond by generating active forces, we can better understand how such active systems evolve and provide insight into emergence of self-organized patterns and functions of living active systems.

## Materials and methods

### Phase-field modeling

In our 2D phase-field model, two order parameters, myosin density c(x,t) and focal adhesion density ρ(x,t), are used to describe the molecular flows, and the displacement field u(x,t) is used to characterize the cell sheet deformation. Following the general framework of the phase-field method, chemical potentials of myosin motor proteins and integrins are derived from the free energy functional, and the chemical potential gradients drive the diffusive motions of the proteins. This leads to two kinetic equations governing the transport of motor proteins and integrins and the mechanical equilibrium equation of the cell sheet. Detailed derivations of chemical potentials are documented in the Supplementary material.

The chemical potentials feature chemomechanical coupling, where mechanical gradients contribute to the driving force for the molecular flows, and the molecular flows result in self-assembly of actin cables and focal adhesions that generate active tension. The resulting kinetics equations and the mechanical equilibrium equation are solved using the COMSOL Multiphysics package.

### Cell culture and imaging

MDCK cells were cultured in Dulbecco's modified Eagle's medium-high glucose (DMEM-HG, with L-glutamin and sodium pyruvate; Gibco), supplemented with 10% fetal bovine serum origin (FBS; EU Approved South American; Gibco) and 1% gentamicin reagent solution (10 mg/mL, Gibco). After seeding MDCK cells, the polyacrylamide (PAA) gel film printed with well-defined adhesive fibronectin patterns was maintained in a 37 °C, 5% CO_2_, and 90% humidity incubator and allowed MDCK cells to form multicellular colonies.

For immunostaining, cells were fixed in 4% paraformaldehyde (Sigma), permeabilized with 0.1% Triton X-100, and blocked with 1% bovine serum albumin (BSA, diluted in deionized water). The samples were stained with Alexa Fluor 488 phalloidin (Thermo Fisher Scientific) for F-actin visualization and DAPI (Thermo Fisher Scientific) for analysis of the cell nuclei. Images were taken using a confocal fluorescence microscope (Leica DMi8) and processed in ImageJ.

### Data analysis and statistics

The local curvature for one piece of actin cable and focal adhesion was obtained by drawing segmented lines along the outer edge of actin cable and focal adhesion cluster, followed by circular fitting of the segmented lines. Afterwards, the mean fluorescent intensity (MFI) of actin signal in multicellular colony was determined for the normalization of local actin cable intensity. Subsequently, the number of focal adhesions was manually counted and the focal adhesion density was calculated by dividing the number of focal adhesions by the length of the segment.

## Supplementary Material

pgad237_Supplementary_Data

## Data Availability

All study data are included in the article text and the Supplementary material.
