# Supplementary material for: Physical forces guide curvature sensing and cell migration mode bifurcating
Source: PNAS Nexus. 2023 Aug 1;2(8):pgad237. doi: 10.1093/pnasnexus/pgad237 (PMC10482382; doi:10.1093/pnasnexus/pgad237)
Supplement: pgad237_Supplementary_Data [file pgad237_supplementary_data.docx]

**
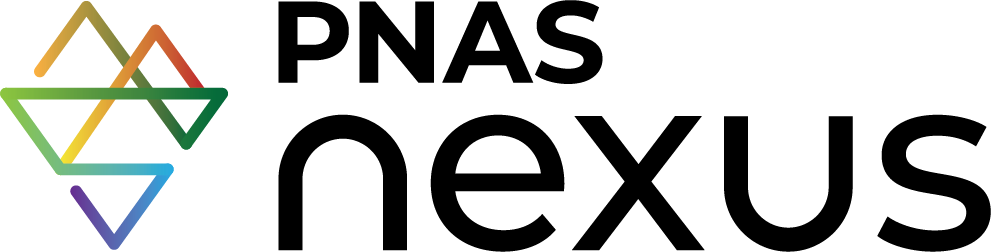
**

**Supplementary Information for**

Physical forces guide curvature sensing and cell migration mode bifurcating

Luyi Feng^1^, Tiankai Zhao^1^, Hongmei Xu^2^, Xuechen Shi^3^, Changhao Li^1^,

K. Jimmy Hsia^2,4^*, and Sulin Zhang^1, 5,6^*

^1^Department of Engineering Science and Mechanics, Pennsylvania State University,

University Park, Pennsylvani­a 16802, United States

^2^School of Mechanical and Aerospace Engineering, Nanyang Technological University, Singapore

^3^Department of Biomedical Engineering, Pennsylvania State University, University Park, Pennsylvania 16802, United States

^4^School of Chemical and Biomedical Engineering, Nanyang Technological University, Singapore

^5^Department of Biomedical Engineering, Pennsylvania State University,

University Park, Pennsylvania 16802, United States

^6^Department of Materials Science and Engineering, Pennsylvania State University,

University Park, Pennsylvania 16802, United States

* To whom correspondence should be addressed: kjhsia@ntu.edu.sg; suz10@psu.edu

**This PDF file includes:**

Supporting text

Figures S1 to S6

SI References

Supporting Information Text

**Phase-field model**

Phase-field model simulations were performed to model the cell as a two-dimensional, active, thin elastic layer. The cell can contract actively, enabled by myosin motors self-assembling actin filaments. The focal adhesions linking cells and substrates are constituted by the complexes between integrin proteins and ligands, sustaining and transmitting traction forces. Therefore, we choose myosin density *c* and focal adhesion density $\rho$ as two order parameters in the phase-field model, and displacement field $\boldsymbol{u}(\boldsymbol{x}, t)$ to characterize the cell sheet deformation out of the intercellular force. We treat the myosin density field as a conserved property, so the temporal and spatial evolution of myosin density is obtained by solving the Cahn-Hilliard equation. The density of the focal adhesion is considered a non-conserved phase variable which is governed by Allen-Cahn equation.

In the phase-field model, the free energy of the cell and the focal adhesion is written as:

$F(c;\rho;\mathbf{u})=\int_{A} [\frac{k_{c}}{2}(\nabla c)^{2}+f_{ch}^{c}(c;\mathbf{u})+f_{el}(c;\mathbf{u})+f_{ch}^{\rho}(\rho;\mathbf{u})]dA+\int_{\partial A} \gamma_{0}d\mathcal{l}$, (1)

where *c* and $\rho$ are order parameters; $\mathbf{u}$ is the displacement field; $\gamma_{0}$ is the line tension acting on the boundary of the cell. In Eq. 1, the first term in the first integration is the gradient energy density; other terms, $f_{ch}^{c}(c;\mathbf{u})$, $f_{el}(c;\mathbf{u})$ and $f_{ch}^{\rho}(\rho;\mathbf{u})$, represent the local energy density from chemical energy contribution of myosin proteins, the elastic energy density in the cell sheet, and chemical energy from the focal adhesions, respectively. For simplicity, we assume that the displacement in the substrate is so small compared with that in the cell sheet, hence the strain energy in the substrate can be neglected.

We model the actomyosin as a material phase that can diffuse freely in the cell domain, with the energy density $f_{ch}^{c}(c;\mathbf{u})$ given by:

$f_{ch}^{c}(c;\mathbf{u})=\Omega c(1-c)+nRT[c\ln c+(1-c)ln(1-c)]-\alpha(c-c_{0})\sigma_{1}$. (2)

The first two terms in Eq. 2 are the double-well energy function (1), whose profile is controlled by the parameter $\Omega$. The second term represents the mixture entropy of the myosin-motor-rich phase and myosin-motor-free phase, where T is the room temperature, R is the ideal gas constant and n is a relative density of *c*. The last term stands for the binding energy contribution from the actin filament formations. Here, we hypothesize that the myosin motors are mainly attracted by the first principal stress $\sigma_{I}$. By using $\sigma_{I}$, we note that the motor proteins mainly feel the tension in the direction of $\sigma_{I}$, in which actin fibers align (2). Once the cell finds that in some regions tension can be established in certain directions, more motor proteins will flow to those regions because the binding energy between the motor proteins and the actin fibers is lower when the fibers are in tension. The parameter $c_{0}$ is the reference myosin density, and $\alpha$ characterizes the strength of the binding between myosin proteins and stress fibers. Overall, the entropic penalty (second term) is balanced by the stress-related enthalpic term (last term).

The cell is modeled as a thin, linear elastic sheet, and the elastic strain energy density $f_{el}(c;\mathbf{u})$ is written as:

$f_{el}(c;\mathbf{u})=\frac{1}{2}h\sigma_{ij}^{el}(c;\mathbf{u})\varepsilon_{ij}^{el}(c;\mathbf{u})$, (3)

where *h* is the cell thickness. The elastic strain tensor $\varepsilon_{ij}^{el}(c;\mathbf{u})$ is:

$\varepsilon_{ij}^{el}\left( c;\mathbf{u} \right)=\varepsilon_{ij}^{tot}\left( \mathbf{u} \right)-\varepsilon_{ij}^{c}\left( c;\mathbf{u} \right)=\frac{1}{2}\left( u_{i,j}+u_{j,i} \right)+(\varepsilon_{0}+\beta c)\delta_{ij}$, (4)

where the first term is the total strain, and the second term is the active contractility of the cell, resembling thermal cooling. The small parameter $\varepsilon_{0}$ is used as a perturbation and $\beta$ is a parameter meaning the active strain increase per myosin density. By applying Hooke’s law for the plane stress, $\sigma_{ij}^{el}(c;\mathbf{u})$ is given by:

$\sigma_{ij}^{el}\left( c;\mathbf{u} \right)=\frac{Y\left( c \right)}{1-\nu^{2}}[\nu\delta_{ij}\varepsilon_{kk}^{el}+\frac{\left( 1-\nu\right)}{2}(\varepsilon_{ij}^{el}+\varepsilon_{ji}^{el})]$, (5)

where *E* is Young’s modulus which is assumed to be linear dependent on *c*:$Y=Y_{0}+ck_{Y}$, with $Y_{0}$ and $k_{Y}$ being constant. As activated myosin proteins are bounded with actin stress fibers, the higher density of the motors means more stress fibers assembly, making the stiffness larger.

The energy density of focal adhesion $f_{ch}^{\rho}(\rho;\mathbf{u})$ is written as:

$f_{ch}^{\rho}(\rho;\mathbf{u})=N(\rho\mu_{0}+\rho\ln\rho+\frac{1}{2}\rho k\left| \boldsymbol{u} \right|^{2}-\rho\zeta)$, (6)

where $N$ is the lattice number on the cell membrane. The first term in Eq. 6 is the energy of reference configuration, where $\mu_{0}$ is the reference chemical potential. The second term is entropy derived from the ideal solution of gases. The third term is the elastic stretch energy in the ligand-receptor bond which is modeled as linear springs. The constant $k$ is the spring constant. Here we use the approximation $\Delta\mathbf{u}\approx\mathbf{u}$. The last term stands for adhesion energy of integrin proteins. Since integrins are force-sensitive and force-responsive molecules, they can feel the extracellular tension and aggregate in response to the existence of the tension. For the parameter $\zeta$, we take the form $\zeta\sim k\left| \mathbf{u} \right|^{2}$. Therefore, the last two terms become $-\frac{1}{2}\rho k\left| \boldsymbol{u} \right|^{2}$, indicating that the enthalpy decreases with increasing $\rho$, which stands for the clustering of integrin in experimental observation (3, 4). Similar to energy structure to actomyosin, the entropic penalty (second term) and tension-induced enthalpy (last two terms) balance with each other.

The chemical potentials with respect to the myosin proteins and integrins are derived from the partial variation of the free energy functional:

$\mu_{c}=\mu_{c}^{ch}+\mu_{c}^{Y}+\mu_{c}^{\sigma}$ (7)

where $\mu_{c}^{ch}=-k_{c}\nabla^{2}c+\left[ \Omega\left( 1-2c \right)+nRTln\frac{c}{1-c} \right]$; $\mu_{c}^{Y}=\frac{{hk}_{Y}}{2Y(c)}\sigma_{ij}^{el}\varepsilon_{ij}^{el}$; and ${\mu_{c}^{\sigma}=h\beta\sigma}_{kk}-\alpha\sigma_{1}$.

$\mu_{\rho}=\mu_{\rho}^{ch}+\mu_{\rho}^{T}$ (8)

where $\mu_{\rho}^{ch}=N(\mu_{0}+1+\ln\rho)$, and $\mu_{\rho}^{T}=(\frac{1}{2}k\left| \boldsymbol{u} \right|^{2}-\zeta)$.

As a molecular whose quantity conserves locally, Cahn-Hilliard equation is adopted to characterize the kinetics of myosin motor evolution:

$\frac{\partial c}{\partial t}=\nabla\cdot M_{c}\nabla\mu_{c}$, (9)

where $M_{c}$ is the mobility parameter. The boundary condition is assumed to be:

$\nabla c\cdot\mathbf{n}=0$, (10)

$\nabla\mu_{c}\cdot\mathbf{n}=0$, (11)

where $\mathbf{n}$ is the outer unit normal vector of the cell boundary.

Since the focal adhesions can be assembled and disassembled, their quantity does not conserve. The kinetics is simply described by Allen-Cahn equation:

$\frac{\partial\rho}{\partial t}=-M_{\rho}\mu_{\rho}$, (12)

where $M_{\rho}$ is the mobility parameter.

The mechanical equilibrium equation and its boundary condition are given by:

$\nabla\cdot(h\boldsymbol{\sigma}\mathbf{)}-\boldsymbol{T}=0$, in $A$, (13)

$\boldsymbol{\sigma}\cdot\boldsymbol{n}=-\gamma\kappa/h \boldsymbol{n}$, on $\partial A$, (14)

where $\boldsymbol{T}=N\rho k\boldsymbol{u}$, derived from interactions between substrates and integrins. The parameters $\kappa$ and **n** are curvature and the outer unit normal of the cell boundary, respectively.

As for the intercellular interaction, we added another boundary condition between two cells to implement adherens junctions.

$\boldsymbol{\sigma}\cdot\boldsymbol{n}=-\tau\Delta\mathbf{u}$, on $\partial_{junction}A$ (12)

where $\tau$ is the stiffness of cell-cell adherens junctions, $\Delta\mathbf{u}$ is the differences on displacement field between two adjacent cells.

**The fluorescent images processing**

**Cell culture and imaging**

MDCK cells were cultured in Dulbecco’s modified Eagle’s medium - high Glucose (DMEM-HG; with L-Glutamin and Sodium Pyruvate; Gibco), supplemented with 10% fetal bovine serum origin (FBS; EU Approved South American; Gibco), and 1% Gentamicin Reagent Solution (10mg/ml, Gibco). Polyacrylamide (PAA) solution was prepared by the mixture of 8% acrylamide and 0.13% bis-acrylamide (Bio-Rad). To prepare PAA gels with a resulting stiffness of 10 kPa, 1/200 total volume of ammonium persulfate (APS, Bio-Rad) and 1/1000 total volume of tetra-methylethylenediamine (TEMED, Bio-Rad) were added to induce free radical polymerization, and 20 µmol/ml 6-((Acryloyl)-amino) hexanoic acid (N-6) (Life Technologies) was included to form binding ligands with fibronectin. Afterwards, 25 µL pre-polymerization solution was sandwiched between an amino-silanated 22×22 mm coverslip and a hydrophobic coverslip to form a thin hydrogel film. The fabricated substrates were immersed in 20 μg/mL human plasma fibronectin (Sigma Aldrich) for one hour and subsequently MDCK cells were seeded. The fibronectin was uniformly coated on the gel surface without patterning. The sample was maintained in a 37°C, 5% CO2 and 90% humidity incubator to allow MDCK cells to form multicellular colonies.

For immunostaining, cells were fixed in 4% paraformaldehyde (Sigma), permeabilized with 0.1% Triton X-100, and blocked with 1% Bovine Serum Albumin (BSA; diluted in DI water). The samples were stained with AlexFluor488 phalloidin (ThermoFisher Scientific) for F-actin visualization and DAPI (ThermoFisher Scientific) for analysis of the cell nuclei. Images were taken using a confocal fluorescence microscope (Leica DMi8) and processed using ImageJ.

The fluorescent image preprocessing was done by Image J Software (Fiji is just Image J) to subtract the background, reduce noise, and refine the detection.

**Actin cable intensity**

The mean fluorescent intensity (MFI) of the actin signal in the multicellular colony is firstly determined for the normalization of local actin cable intensity. Drawing an outline along the desired cell area as the region of interest (ROI) using ‘Freehand Selections’ tool on high-resolution tablet. Measure the mean gray value of each pixel in ROI as the average cell colony intensity.

To obtain the local curvature for one piece of actin cable, ‘Segmented line’ tool was employed to draw edgewise along the outer edge of actin cable, followed by circular fitting of the segmented line. The curvature is derived by taking the reciprocal of the radius of curvature of the fitting circle.

To quantify the intensity of one piece of formed actin cable, short straight lines were placed cross curved actin cable. Subsequently, the gray value profile along one piece of line segment was acquired via ‘Plot Profile’ tool. The actin cable region is considered as the pixels lying on the outermost side of the cell colony with the gray value higher than the other part of multicellular colony, and thus the mean gray value of these pixels is deemed as the actin cable intensity. At least five pieces of profiles were taken into the calculation and the statistical average of gray value was considered as the actin cable intensity.

To make a comparison between different frames of cell colonies, the actin cable intensity was further normalized by the MFI of multicellular area.

**Focal Adhesions (FAs) density**

The line segments were drawn along the focal adhesion clusters, and a similar fitting method was adopted to derive the curvature.

The number of focal adhesions was manually counted. Subsequently, the FAs density was calculated by dividing the number of focal adhesions by the length of the segment.

Fig. S1.


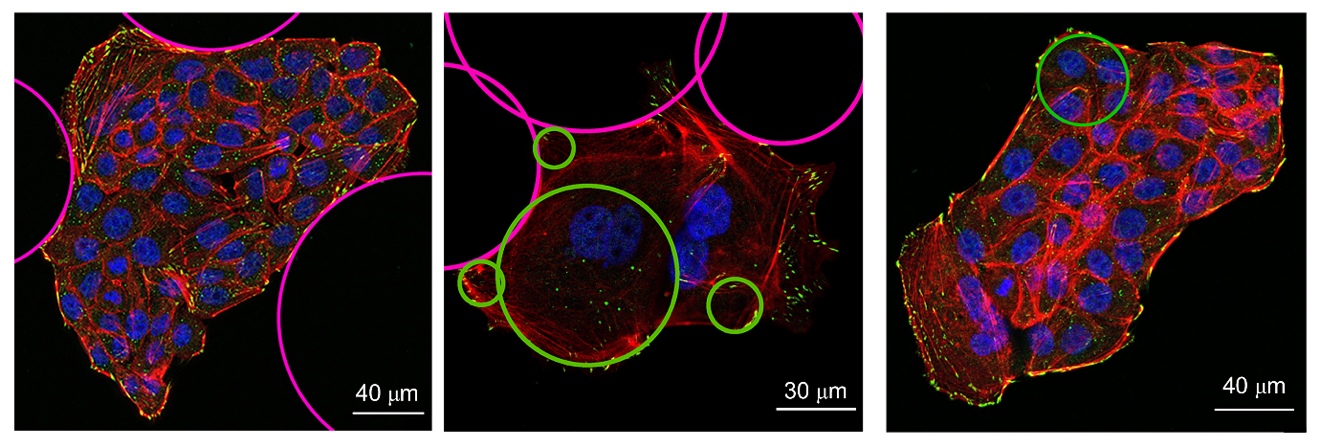


Fig. S1. Experimental measurements of the density of the actin stress fibers (concave fronts, curvature measured with purple circles) and focal adhesions (convex fronts, curvature measured with green circles), corresponding to the cell front curvatures

**Fig. S2.**


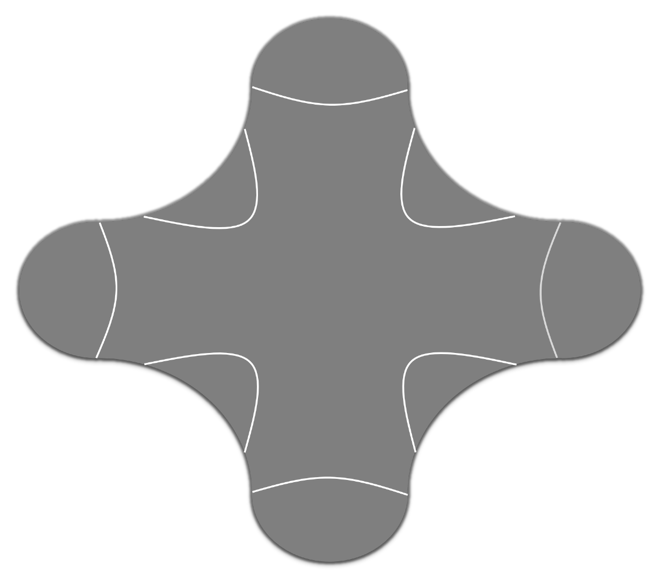


**Fig. S2.** Our simulation model consists of a flower-like multicellular colony with differently curved front edges. In the model, line tension-induced flows of actomyosin motors and integrins are considered for the self-assembly of actin cables and focal adhesions at the curved front. For the concave and convex domains divided by white lines, the length of front line and domain area are controlled as same.

Fig. S3.


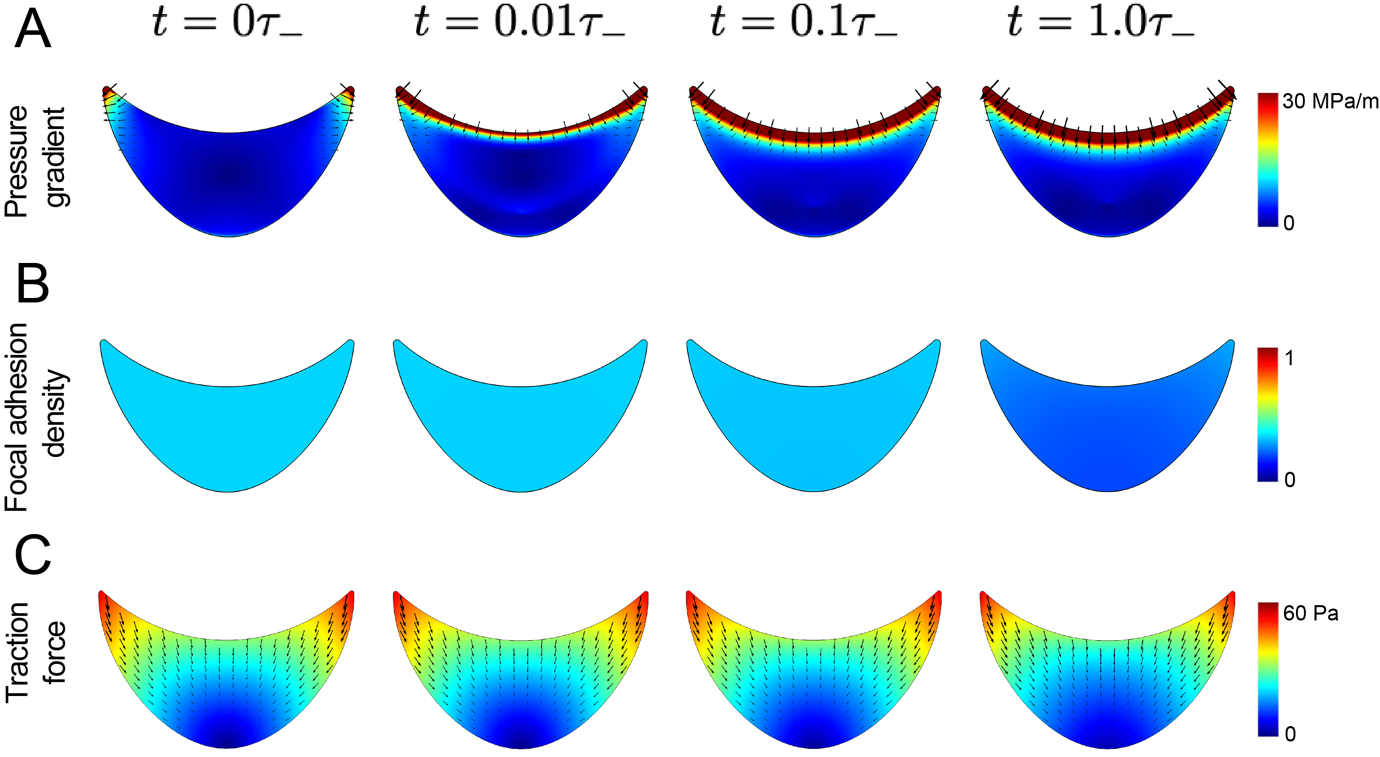


**Fig. S3** **A**. The pressure gradient induced by line tension is amplified due to the actomyosin motor flow toward the concavely curved front and actin cable formation. **B**. The integrin flow is nearly inactive and the integrin distribution is almost uniform overall in the cell domain. **C**. The traction force changes little during the evolution, and points in the inward direction, acting like a frictional force.

Fig. S4.


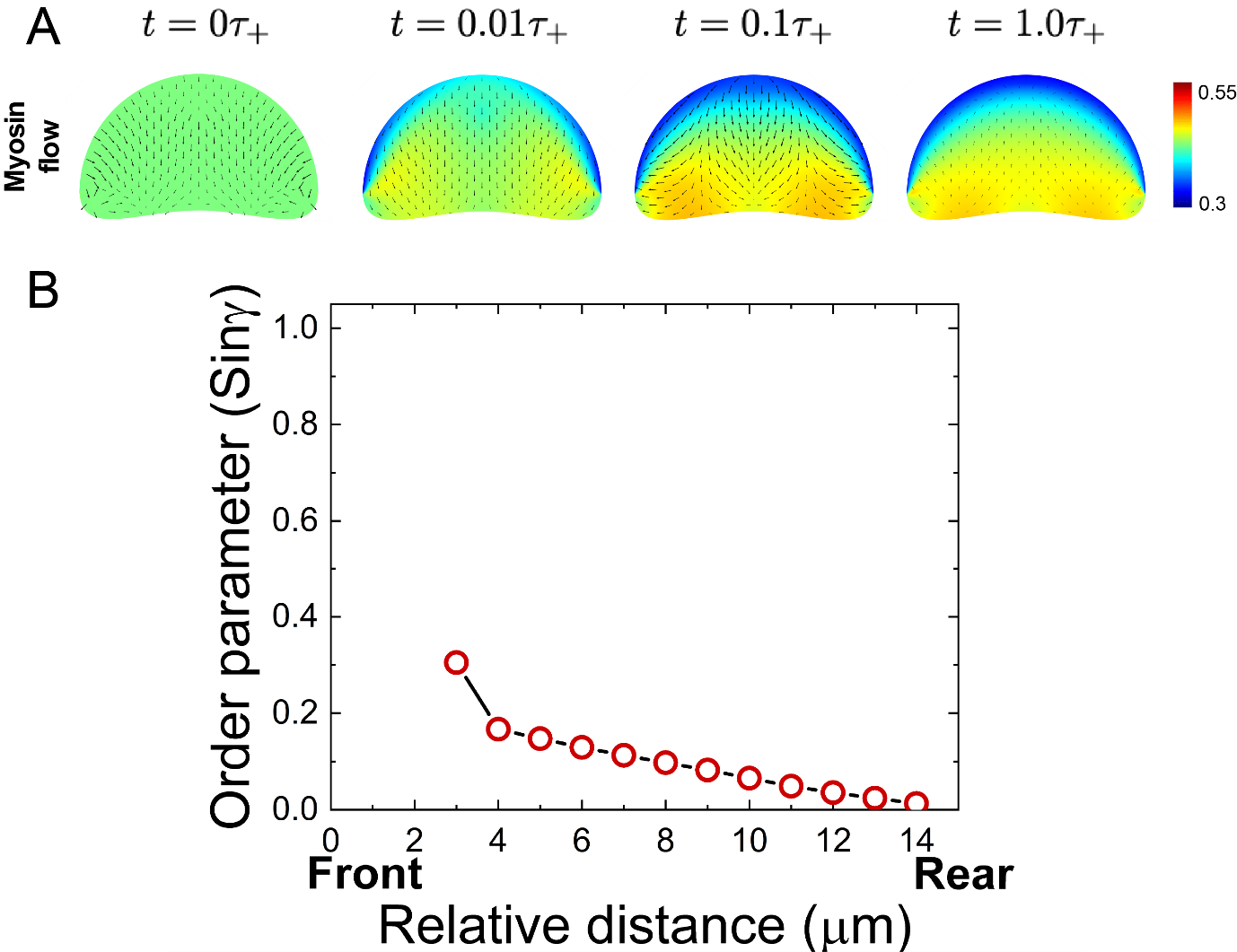


**Fig. S4** Front-to-rear actomyosin flow in the cell cytosol is activated at the convexly curved front (**A**), resulting in a stress anisotropy indicated by the order parameter $\sin\gamma$ **(B)**. γ is the angle of actomyosin flow with respect to the forward direction. $\sin\gamma$ approaches 0 means actin cables are aligned from rear to front, while $\sin\gamma$ approaches 1 means actin cables are perpendicular to the forward direction.

Fig. S5.


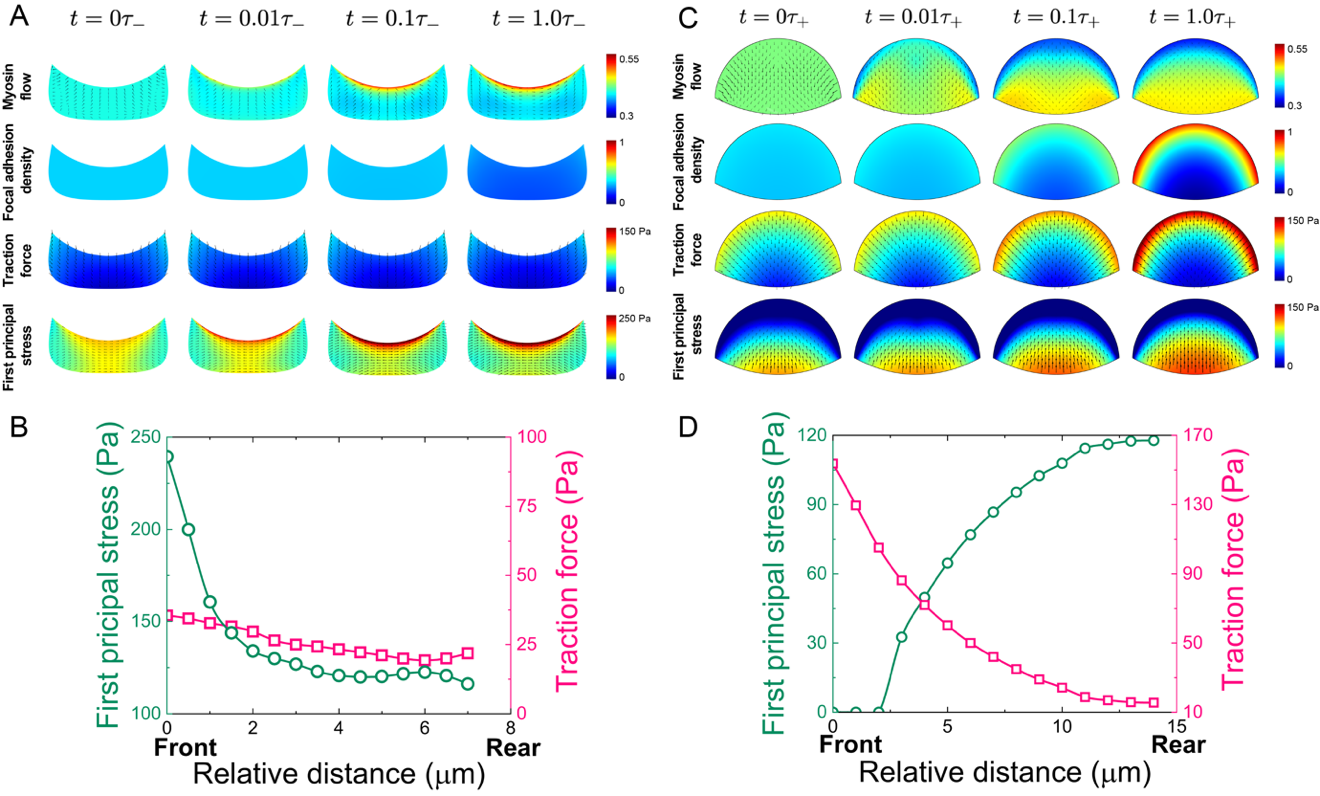


**Fig. S5** The feedback loops trigged by the line tension at the curved fronts are weakly dependent on the rear-side shape of the cells. The resulting cellular force distributions are nearly the same, as shown in Fig. 3 of the main text. **A-B**: A cell with the same concave front edge as in Fig. 3A but different rear-side shapes. **C-D**: A cell with the same convex front edge in Fig. 3C but different rear-side shapes.

Fig. S6.


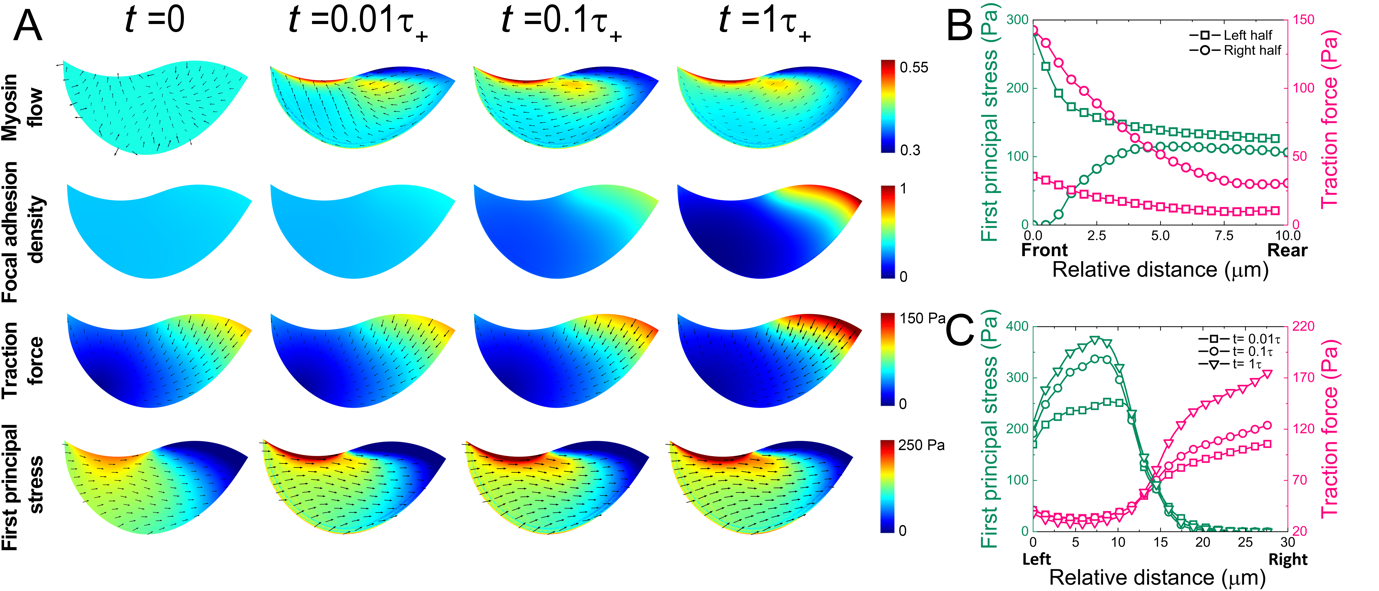


**Fig. S6** Curvature sensing and migration-mode bifurcation for cells with transitional curvatures. **A**. Interdiffusions of actomyosin motor proteins and integrins, leading to self-assembly of actin cables at the concave region and focal adhesions at the convex region, with a smooth transition in between. Accordingly, cell stress and traction force landscape also exhibit a transition behavior at the migration front. **B**. Left-right symmetry breaking at the curve front, manifested by the traction force and first-principal stress. **C**. Front-rear symmetry breaking manifested by the traction force and first-principal stress.

**SI References**

1. L. Chen *et al.*, A phase-field model coupled with large elasto-plastic deformation: application to lithiated silicon electrodes. *Journal of The Electrochemical Society* **161**, F3164 (2014).

2. T. Chen *et al.*, Large-scale curvature sensing by directional actin flow drives cellular migration mode switching. *Nature Physics* **15**, 393-402 (2019).

3. F. Lu *et al.*, Mechanism of integrin activation by talin and its cooperation with kindlin. *Nature Communications* **13**, 2362 (2022).

4. K. Chinthalapudi, E. S. Rangarajan, T. Izard, The interaction of talin with the cell membrane is essential for integrin activation and focal adhesion formation. *Proceedings of the National Academy of Sciences* **115**, 10339-10344 (2018).
